# Method for organic fluorine determination in gasoline and its components using high-resolution continuum source flame molecular absorption spectrometry with gallium fluoride as a target molecule

**DOI:** 10.1016/j.mex.2021.101564

**Published:** 2021-10-29

**Authors:** Zofia Kowalewska, Karolina Brzezińska, Janusz Zieliński, Janusz Pilarczyk

**Affiliations:** a Faculty of Civil Engineering, Mechanics and Petrochemistry, Warsaw University of Technology, Łukasiewicza 17, Płock 09-400, Poland; b WARTER Fuels JSC, Chemików 5, Płock 09-411, Poland

**Keywords:** F determination, HR-CS, HR-CS MAS, HR-CS FMAS, GaF, Organic solution

## Abstract

Organic fluorides can arise due to the usage of HF in technologies of gasoline production, therefore, it is necessary to identify contamination of gasoline or its components with organic fluorine. For this aim, simple and fast method is proposed. The method relies on sample dilution in xylene, followed by the solution aspiration to a high-resolution continuous source spectrometer for measurement of absorption of radiation by GaF molecule, arisen in air-acetylene flame. Gallium(III) acetylacetonate is carefully dissolved and introduced to all measured solutions at Ga concentration 5000 mg L^−1^. For standard addition calibration three types of samples solutions are prepared to contain: none, 50 mg L^−1^ and 100 mg L^−1^ of added fluorine. As fluorine standard 2, 2, 3, 3, 4, 4, 4-heptafluoro-1-pentanol is applied. The lowest possible flow rate of acetylene is recommended. The solution flow rate and the additional air flow rate should be adjusted to obtain not-disturbed baseline. The overlap of the GaF signal with residual signal of the OH molecule can be overcome using least square background correction. Five pixels are recommended for signal evaluation at the most sensitive 211.248 nm rotational “line”. Using such conditions characteristic concentration was 3.2 mg L^−1^. Detection limit recalculated for initial sample was 4-10 mg L^−1^.

• High-resolution continuum source flame molecular absorption spectrometry turned out to be an excellent tool for determination of pollution of gasoline with organic fluorine.
• Due to application of GaF as a target molecule, it is possible to use a low-temperature and easy for operation air-acetylene flame.
• The proposed method can be applied for analysis of alkylate, reformate, isomerizate, ethanol as well as commercial automotive and aviation gasoline.

## Graphical abstract

**Figure:**
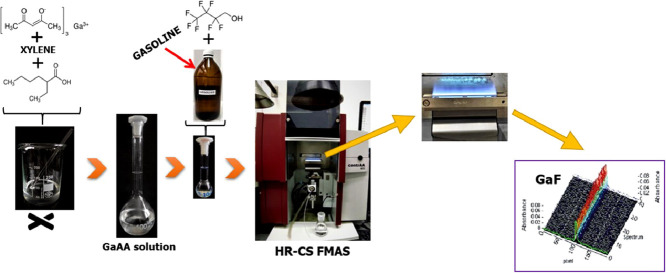

Specifications table

**Table:** 

Subject Area	Chemistry
More specific subject area	Analytical chemistry; Analytical spectrometry
Method name	Organic fluorine determination in gasoline and its components using high-resolution continuum source flame molecular absorption spectrometry with gallium fluoride as a target molecule
Name and reference of original method	Method development for determination of organic fluorine in gasoline and its components using high-resolution continuum source flame molecular absorption spectrometry with gallium fluoride as a target molecule, 10.1016/j.talanta.2021.122205 (Co-submission)
Resource availability N.A.	

## Method details

### Scope, principle and background of the method

The aim of this paper is presentation of a method devoted to organic fluorine determination in gasoline and its components. The development of the method and its scientific background was presented in detail in a paper published alongside this work in Talanta [1]. The method relies on sample dilution in xylene, with addition of necessary reagents and standards and aspiration of the solutions into flame of high-resolution continuous source absorption spectrometer. Simple, low temperature and easy for operation air-acetylene flame is used to decompose organic fluorine compounds as well as gallium(III acetylacetonate, applied as a source of gallium. If fluorine is present in solution, gallium monofluoride is formed in the flame, which is used as a target molecule, absorbing radiation at selected, specific wavelength. The absorption is due to changes of rotational states of the molecule. The measured most sensitive rotational “line” at 211.248 nm belongs to the ∆ν = 0 vibrational transmission within the X^1^Σ^+^→C^1^П electronic transition. As gallium is added in huge and the same amount to all working solutions, the measured absorption by GaF molecule is directly proportional to the fluorine content in the investigated working solutions. Unfortunately, only one compound of Ga, dissoluble in xylene and samples, was available, gallium(III) acetylacetonate. Its dissolution is not easy and should be carried out with care.

High resolution continuum source molecular spectrometry, known in its commercial version since the first decade of this century, is widely applied for fluorine determination [2], [3], [4], [5]. However, almost all papers concern electrothermal version with target molecules generated in a graphite furnace (the technique is very sensitive, but slow). What is more, none work is known on determination of fluorine in gasoline or similar materials using both graphite furnace and flame molecular absorption techniques. At the same time flame atomic absorption spectrometry is widely applied in petroleum laboratories, also for analysis of gasoline [6], [7], [8]. If a laboratory uses atomic absorption spectrometer in the high-resolution continuum source version, there is the possibility to widen the equipment application range for determination of fluorine, according to the proposed method, by measurement of absorption by the GaF molecule.

Due to relatively low sensitivity of the flame technique, to achieve the best possible detectability, it is beneficial to dilute sample only at the lowest possible degree. Unfortunately, in such a case, sample composition can influence measurement result. Various gasoline components and various gasoline grades can differ significantly in their composition and properties [1,6]. Physical properties, such as surface tension, density and viscosity will influence even solution aspiration. Influence of sample composition on sample combustion also can be very important as the aspirated solution containing diluted sample is an additional fuel for flame [1,6]. In the proposed method a few strong tools are available to overcome the problems. Very noisy background formed in the case of too rich flame can be minimized/eliminated by application of minimum acetylene flow rate as well as by adjusting additional air flow rate and solution aspiration rate. For background correction, iterative mode can be applied (iterative background correction), based on absorption minima [2]. The correction enables effective elimination of continuous events as both target absorption and background are measured simultaneously [2]. Additionally, sophisticated mode of least square background correction can be used for elimination of any residual effect of OH molecule (the spectrum of the OH molecule partially overlaps measured range of absorption by the GaF molecule at the 211.248 nm rotational “line”) [1].

Another aspect of the work is selection of standard. On the basis of technological data, it is expected that fluorine is present in investigated samples in forms of low molecular mass and high volatility organic compounds [1]. This kind of compounds should give relatively high signal in the applied method. At the method development step 2, 2, 3, 3, 4, 4, 4-heptafluoro-1-butanol has been proposed as a standard, because it gave the highest signal. It is also cheap, easily available, and easy to handle. The choice of the standard was confirmed [1] in comparative analysis with routine combustion ion chromatography [9] as a reference method.

The proposed HR-CS FMAS method was applied for the following materials: alkylate, reformate, isomerizate, automotive gasoline, leaded aviation gasoline, unleaded aviation gasoline and ethanol [1]. Using sample dilution ratio 1:10, v:v, the detection limit recalculated for initial (original) sample is 10 mg L^−1^ and the determination limit is 31 mg L^−1^. This is the analysis mode obligatory for highly volatile samples as isomerizate. In the case of other investigated samples more beneficial dilution ratio 1:4, v:v was possible and the detection limit recalculated for the initial sample was 4 mg L^−1^, while the determination limit was 12 mg L^−1^. The upper limit of the range of the method is at least 100 mg L^−1^ in solution, that is 400/1000 mg L^−1^ in the initial sample. The method serves to detect contamination of components considered for gasoline blending or contamination of produced gasoline with organic fluorine. It is an important issue as organic fluorine constitutes significant corrosion and environmental threat.

**Fig. 1:**
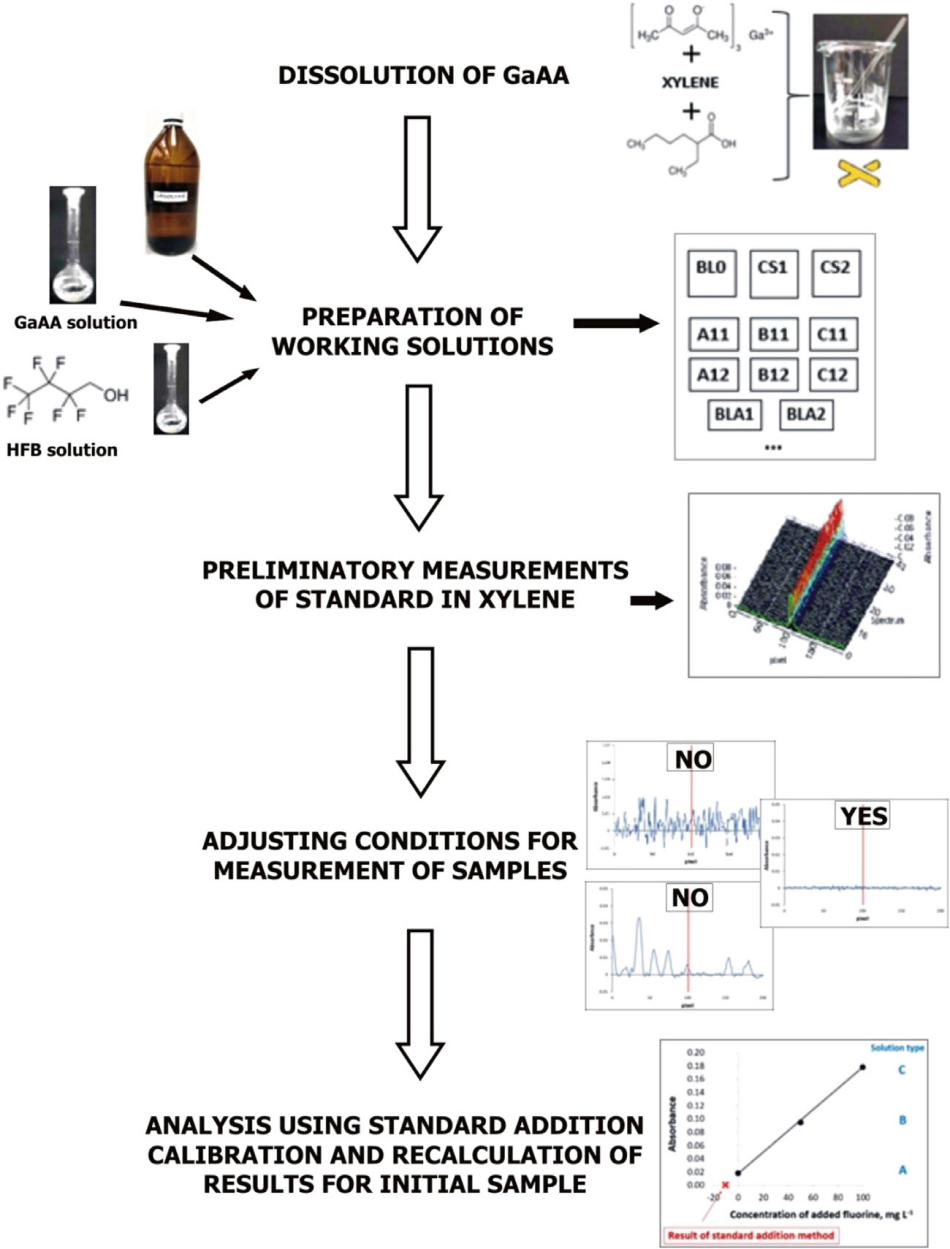
Flowchart of the proposed method.

The flowchart of the proposed method is presented in Fig. 1.

All work according to the proposed procedure should be carried out in compliance with requirements and guidelines of producers of the used equipment as well as in compliance with health and safety regulations concerning used chemicals/materials.

### Instrumentation and laboratory equipment

In this work commercially available high-resolution continuum source absorption spectrometer contrAA 700 (Analytik Jena A.G., Jena, Germany) was used for organic fluorine determination. The spectrometer was equipped with a high-intensity xenon short-arc lamp, a high-resolution monochromator (consisting of a prism and an echelle grating) and a linear charge-coupled device array detector with 200 pixels available for analytical purposes. The apparatus worked in high-resolution continuum source flame molecular spectrometry (HR-CS FMAS) mode. The flame system was resistant to both organic solvents and hydrogen fluoride. An air-acetylene flame and a burner with a 100 mm slit were used.

Equipment needed for preparation of solutions of samples and standards:

- analytical balance,
- electric cooker,
- beakers, 150 mL,
- graduated glass flasks, 10, 25, and 100 mL,
- Erlenmeyer flasks, 100, and 250 mL,
- graduated glass pipets, 1,0, and 5,0 mL,
- glass rode for stirring,
- graduated glass cylinder,
- plastic pipettes of Pasteur type

### Reagents

- 2, 2, 3, 3, 4, 4, 4-heptafluoro-1-butanol (HFB), 66.44% F from Sigma Aldrich (Germany),
- Ga(III) acetylacetonate (GaAA), 18.99% Ga from Sigma Aldrich (Germany),
- xylene as a mixture of isomers, solvent of analytical grade from POCh, (Gliwice, Poland),
- 2-ethylhexanoic acid from Sigma Aldrich (Germany).

The reagents used in this work were at least of analytical grade.

### Preparation of solutions

*Gallium (as GaAA) basic solution, containing 10 000* mg L^−1^ *of Ga:* To 5.266 g (± 0.001 g) of GaAA, weighted in a 150 mL glass beaker, 65 mL of xylene should be added, and the content of the beaker should be gently heated on an electric cooker and stirred with a glass rod. After 20 min the beaker should be taken of the cooker and cooled, to add 10 mL of 2-ethylhexanoic acid. Next, the heating of the beaker and mixing of its content should start again and be continued until salt dissolution. The cooled solution should be quantitatively transferred to 100 mL volumetric flask. The obtained solution can show some opalescence, but, should not contain any sediment. It is preferable to use this solution on the day of its preparation.

*Remark:*

*The volume of 100 mL of the GaAA solution will enable preparation of all solutions needed for analysis of two samples in duplicate, including samples and standard solutions for standard addition calibration, blank, as well as control solutions. In the case of analysis of more samples, more GaAA should be prepared. To spare GaAA, it can be beneficial to determine final mass of the solution, instead of volume. In this way, any calculated as necessary amount of the GaAA can be prepared, without limitation of available volumetric flasks.*

*Basic solution of fluorine, containing 10,000 mg L^−1^ of F (as HFB):* Approximately 0.301 g (± 0.001 g) of HFB was weighted into a 100 mL Erlemeyer flask and diluted to final mass 50,000 g (± 0.001 g) with xylene.

*Intermediate solution of fluorine, containing 1 000 mg L^−1^ of F (as HFB):* Approximately 5.000 g (± 0.001 g) of basic solution of fluorine, containing 10,000 mg L^−1^ of F (as HFB) was weighted into a 100 mL Erlenmeyer flask and diluted to final mass 50,000 g (± 0.001 g) with xylene.

*Blank sample*: a sample, which does not contain measurable F content. In the best case blank sample is of the type of the investigated samples.

Working solutions comprise solutions listed below. Details of their preparation can be found in Table 1.

**Table 1:** Preparation of working solutions.

Target solution	Volume of investigated sample, mL	Volume of blank sample, mL	Mass of Ga 10 000 mg L^−1^ solution, g	Mass of intermediate F solution, containing 1000 mg kg^−1^ F, g	Final volume of solution, mL
Sample solution of type A (no added F)	1.0	0	4.4	-	10
Sample solution of type B (50 mg L^−1^ of added F)	1.0	0	4.4	0.5	10
Sample solution of type C (100 mg L^−1^ of added F)	1.0	0	4.4	1.0	10
Blank solution, BL0	0	2.5	0	0	25
Blank solution of type BLA	0	1.0	4.4	0	10
Control solution 1, CS1, containing 100 mg L^−1^ F	0	0	11.0	2.5	25
Control solution 2, CS2, containing 100 mg L^−1^ F and blank sample	0	2.5	11.0	2.5	25

*Blank solution, BL0*: a solution containing blank sample, diluted 1:10 v:v in xylene (without GaAA).

*Blank solution, BLA*: a solution containing blank sample, prepared as investigated samples of type A (with GaAA).

*Control solution 1, CS1*: a solution containing 100 mg L^−1^ of F (as HFB) and GaAA in xylene.

*Control solution 2, CS2,* a solution containing 100 mg L^−1^ of F (as HFB), GaAA and blank sample (diluted 1:10 v:v) in xylene.

*Sample solution of type A*: a solution containing the investigated sample (diluted 1:10 v:v) and GaAA, without added F.

*Sample solution of type B*: a solution containing the investigated sample (diluted 1:10 v:v), GaAA and 50 mg L^−1^ of added F (as HFB).

*Sample solution of type C*: a solution containing the investigated sample (diluted 1:10 v:v), GaAA and 100 mg L^−1^ of added F (as HFB).

The content of Ga in working solutions, quoted above and in Table 1, was equal to 5000 mg L^−1^, with the exception of BL0. The BL0 solution is used to adjust and control baseline. As the presence of Ga in the solution is not indispensable, it was not added (saving of GaAA).

Solutions A-C of a given sample are for standard addition calibration. The solutions should be prepared in duplicate. Thus, for a given sample of number 1 the working solutions can be labelled as A11, B11, C11, A12, B12 and C12. For each series of samples, two solutions of blank of type BLA should be prepared (BLA1 and BLA2).

## Performing analysis, calculation of results

### Switching apparatus and preliminary measurements of standard in xylene

Spectrometer should be operated according to requirements and recommendations of its producer, including operation of flame system during analysis with an organic solvent. In the case of necessity of solvents of various polarity changes, for example, in the case of switching between water and xylene, isopropanol can be used as an intermediate solvent.

The flame system should be washed before analysis, firstly with isopropanol and finally with xylene. ContrAA spectrometer does not require preheating of xenon lamp and analysis can start just after a few minutes since flame ignition (burner heating).

Operating conditions of spectrometer are given in Table 2.

**Table 2:** Working parameters of spectrometer.

Parameter, unit	Parameter value
Burner type, mm	100
Burner angle, deg	0
Flame type	Air-C_2_H_2_
Acetylene flow rate, L h^−1^	40
Additional air flow rate, L h^−1^	75-150
Observation height, mm	6
Solution aspiration flow rate, ml min^−1^	4-5
Delay time, s	8
Measurement (read) time, s	3
Pre-run	1
Replicates of measurement of each solution	3
Wavelength, nm	211.2480
Spectral range, nm	211.1230–211.3718
Spectral range, pixels	0-200
Number of pixels taken for signal evaluation	5
Numbers of measured pixels	99-103
Background correction	IBC*+ LSBC**
Permanent structures correction	off

*iterative background correction

**least square background correction

Flame should be ignited, when no solution is aspirated, the acetylene flow rate is relatively high, for example, 50 L h^−1^, and the additional air is not applied. Only when the flame gets stable and lightly green, the aspiration capillary can be introduced to xylene and, immediately, the acetylene flow rate should be decreased to the lowest rate of 40 L h^−1^ as well as the additional air flow rate should be set to 75 L h^−1^. Xylene aspiration rate should be measured (e.g. using glass cylinder of 10–20 mL and stopwatch) and adjusted to approximately 5 mL min^−1^.

It is necessary to assure continuous delivery and aspiration of xylene or working solutions for the whole analysis time, because the solutions are fuel for flame. Lack of delivery of organic solution means too low amount of fuel for keeping the flame and may result in flashback. The breaks necessary to bring an aspiration capillary from one solution to another solution should be as short as possible. The spectrometer should not be left alone.

**Fig. 2:**
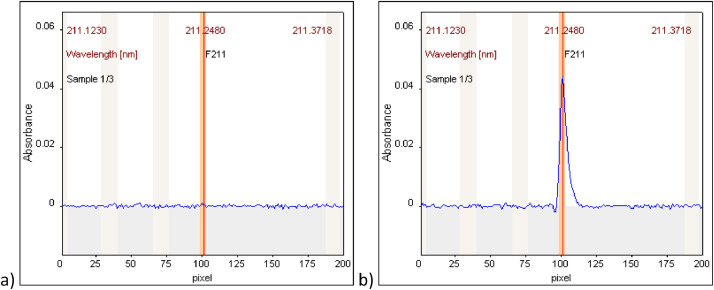
Example spectra obtained for: xylene (a), 100 mg L^−1^ F and 5000 mg L^−1^ Ga in xylene (b).

**Fig. 3:**
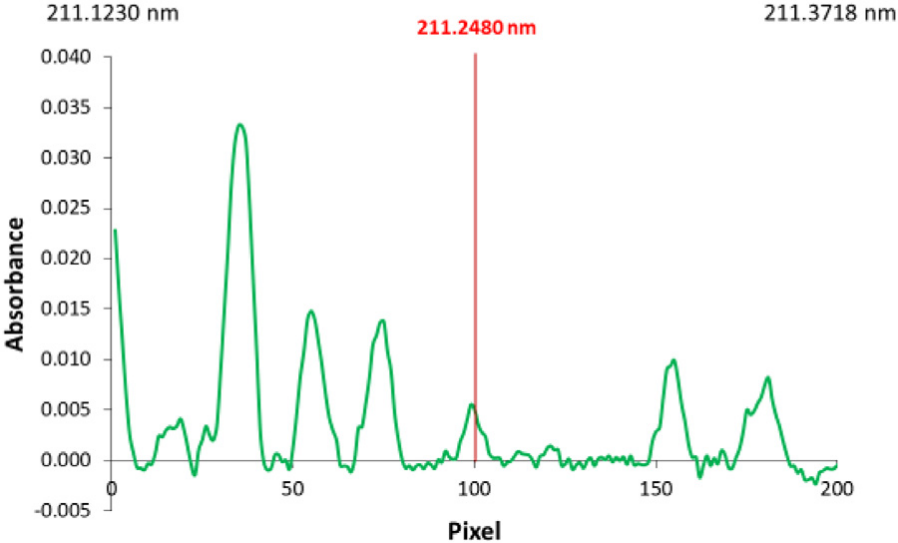
Example spectrum showing non-compensated OH molecule absorption.

For introductory checking of the proper work of apparatus, before start of analysis, it is advisable to measure CS1 solution, which contains only Ga (GaAA) and F (HFB) in xylene, without any sample addition. For that, flame optimization window in a manual version should be opened (in the current case of organic solutions analysis, it is not recommended to use flame automatic optimization). After setting the required wavelength of 211.2480 nm and starting measurement, a baseline can be observed, related to the aspirated xylene. Using the above listed conditions, a stable baseline should be obtained. An example is presented in Fig. 2a (another example can be seen in Fig. 3a from the reference paper [1]). Then, control solution, CS1, containing 100 mg L^−1^ of F as well as 5000 mg L^−1^ of Ga can be aspirated and the GaF molecule should be formed in flame. The increase of absorbance to at least 0.13 means that appropriate conditions have been reached for xylene. An example spectrum of CS1 solution is shown in Fig. 2b.

### Adjusting conditions for samples measurements

**Fig. 4:**
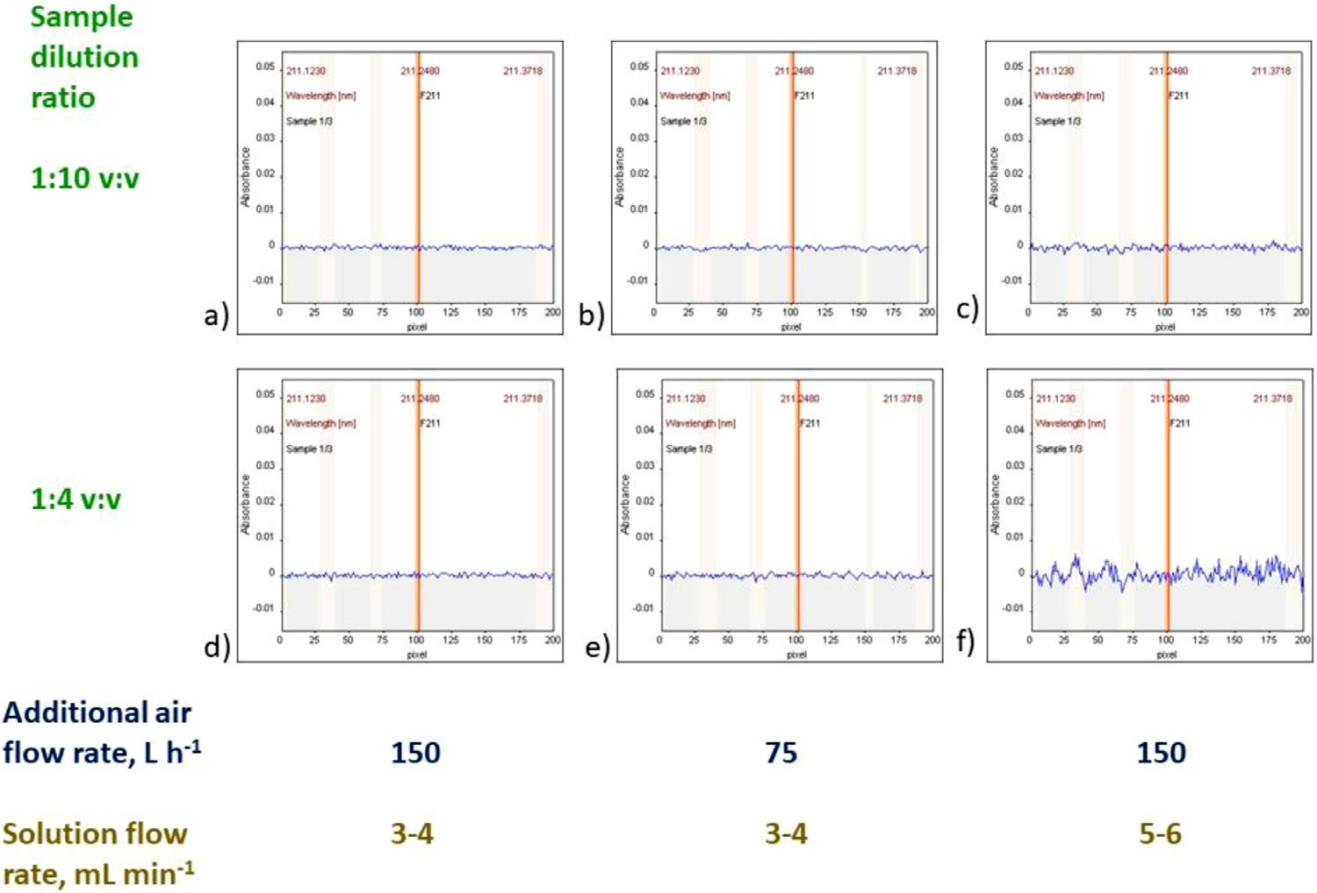
Example signals obtained using various sample dilution ratio and/or solution flow rate and/or additional air flow rate. Sample: alkylate.

**Fig. 5:**
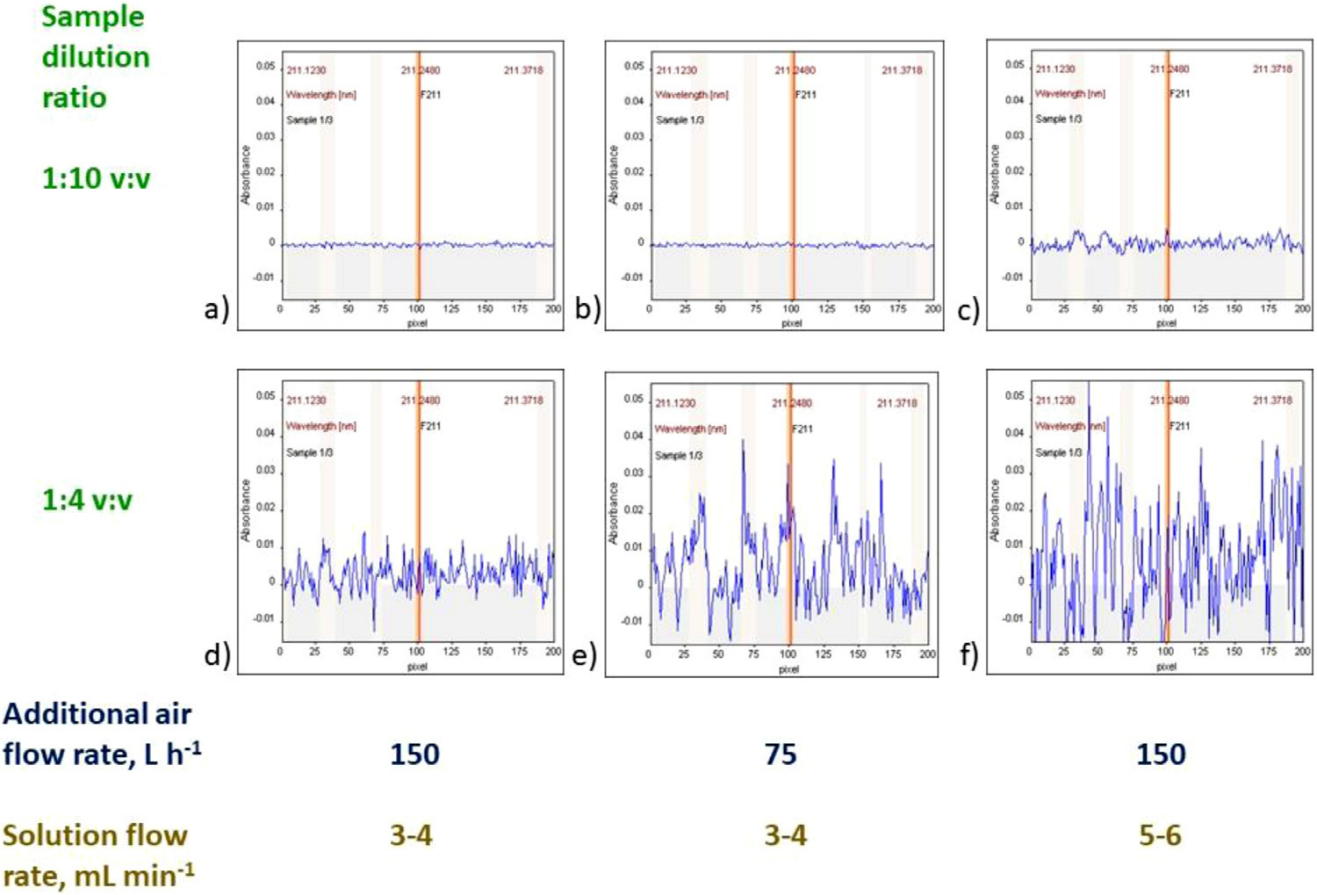
Example signals obtained using various sample dilution ratio and/or solution flow rate and/or additional air flow rate. Sample: isomerizate.

Blank solution BL0 (containing blank sample diluted 1:10 v:v) and control solution 2, CS2 (containing 100 mg L^−1^ of F, 5000 mg L^−1^ Ga and blank sample diluted 1:10 v:v) should be aspirated to establish the best conditions for measurements of samples. It is possible that the presence of sample in the aspirated solution can significantly change the appearance of flame and baseline, as well as can influence the signal for 100 mg L^−1^ of F. It is recommended to adjust conditions to get the best signal for samples solutions. For that, the additional air flow rate should be adjusted to obtain not-disturbed baseline for the blank sample. Too high additional air flow rate means too “lean” flame, which can result in not compensated absorbance by the OH molecule (e.g. Fig 3). The overlap of GaF signal with the residual signal of OH molecule is expected to be overcome using the least square background correction (LSBC). Too high air flow rate can also mean decrease of the GaF signal (see Fig. 2c in [1]). On the other hand, too low additional air flow rate means too “rich” flame, which can cause intensive noise. Such noise can be observed in Figs. 4f, 5c–f (as well as in Fig. 3a in [1]). In the case of severe problems with too “rich” flame, when 150 L h^−1^ of additional air flow rate is not sufficiently high, it is recommended to reduce the solution aspiration rate (below 5 ml min^−1^). It is not recommended to use available option of 225 L h^−1^ of the additional air flow rate due to the risk of flame blowing out.

To execute the LSBC a generation of the OH spectrum is necessary. For that, very lean flame should be set up. It was shown [1] that the OH spectrum was accidentally and spontaneously generated due to stepwise blockage of solution aspiration tube (Fig. A2 in [1]). For the OH spectrum generation it is recommended to set the following conditions: none solution aspiration, 50 L h^−1^ acetylene flow rate and no additional air. A measurement should start and then, acetylene delivery should be shut down. Sudden decrease in fuel amount should result in the OH molecule spectrum generation before the flame is extinguished.

Measurements of samples solutions should be carried out in conditions which assure the best (most stable) baseline in measurement of BL0 as well as the best (the highest) absorbance in measurement of CS2. For most samples (e.g. alkylate, reformate, commercial automotive gasoline [1], aviation leaded gasoline and aviation unleaded gasoline of various types), using dilution ratio 1:10 v:v, it was not necessary to introduce any changes of conditions in comparison with the xylene measurements. This is due to relatively high dilution ratio. At the same time, standard addition method applied here for calibration enables to overcome differences in physical properties of various samples, such as boiling point, surface tension, viscosity, boiling range, density and vapour pressure.

Moreover, for relatively low volatility samples, such as alkylate, reformate, Avgas 100 LL gasoline, various unleaded aviation gasolines and automotive gasoline, it was possible to lead measurements using lower dilution ratio, 1:4 v:v. In this way significant improvement of detection limit, from 10 to 4 mg L^−1^ of F in recalculation for the original sample, was possible.

In the case of aspiration of very volatile samples as isomerizate there is a high risk of too rich flame and dilution ratio 1:10 v:v is recommended. Additionally, some modification of conditions can be necessary (an increase of the additional air flow rate or a decrease of the solution aspiration rate).

Some examples of signals are presented in Figs. 4,5, obtained, respectively for an alkylate (which represents medium volatility samples) and an isomerizate (which represents high volatility samples). The registered baseline is sufficiently stable in most measurements of alkylate (Fig. 4a–e) and only in some cases of measurements of isomerizate (Fig. 5a,b).

### Analysis, calculation and expression of results

The adjusted conditions should be stored and applied. Each analysis sequence should start with reference measurement for optics setup. During this measurement, xylene should be aspirated. For control of stability of flame, it is recommended to check flame stoichiometry by observation of flame appearance during periodic aspiration of BL0 solution. A symptom of blockage of solution introduction system would be more “lean” flame (flame getting blue). For more precise control of sensitivity, the CS2 (or the CS1) solution can be measured from time to time (e.g. between various samples or every ten solutions) and its absorbance should be stable within ± 5%.

**Fig. 6:**
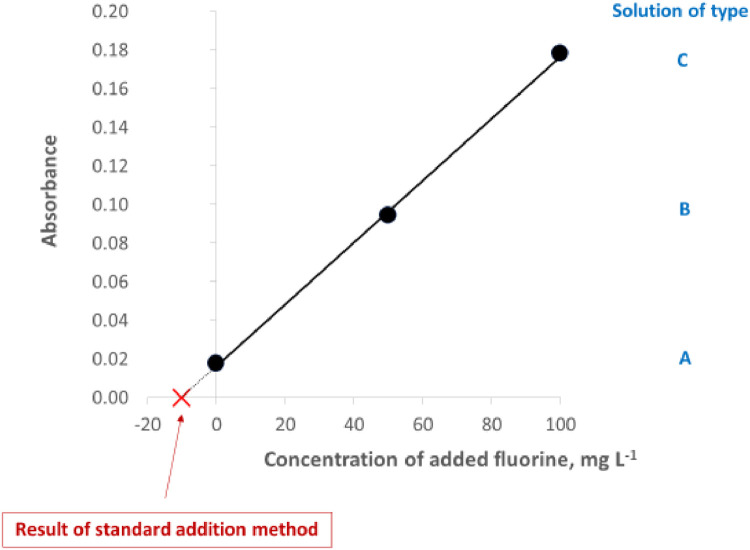
Example plot in standard addition method used for calibration.

For each sample, series of A-C solutions should be measured at least in duplicate. For solutions A1-C1 (as well as for solutions A2-C2) results of measurements of absorption are used to obtain plots of standard addition method. The content of F in a sample solution of type A is found from such kind of plot by extrapolation. An example is shown in Fig. 6. The correlation coefficient of curve in standard addition calibration should be better than 0.995. Example and detailed data of standard addition method for a few alkylate samples are given in Table 3, while Table 4 contains data for other kinds of samples. It should be added that various samples could have been analyzed on various days, using slightly various conditions, which could have caused some differences in sensitivity.

**Table 3:** Example data of standard addition method for alkylate samples.

Name of solution	Average absorbance for solution (RSD of measurements, %)	Equation of curve in standards addition method (correlation coefficient of the curve)	Result of standard addition method, mg L^−1^	Sample dilution ratio, v:v	Result recalculated for initial sample, mg L^−1^	Final result for sample, mg L^−1^
Alkylate 1						
A11	0.0235 (10.1)					
B11	0.0941 (2.5)					
C11	0.1690 (4.2)	y=0.00146x+0.02278 (0.9998)	15.6	1:4	62	
A12	0.0204 (6.0)					
B12	0.0847 (1.1)					
C12	0.1570 (1.2)	y=0.00137x+0.02007 (1.0000)	14.7	1:4	59	61 ± 4*
Alkalyte 2						
A21	0.00584 (3.6)					
B21	0.08234 (2.8)					
C21	0.16784 (1.4)	y=0.00155x+0.0551 (1.0000)	3.6	1:10	36	
A22	0.00437 (5.0)					
B22	0.08727 (4.6)					
C22	0.15701 (5.9)	y=0.00153x+0.0657 (0.9987)	4.3	1:10	43	40 ± 7*
Alkylate 3						
A11	0.00012 (220)					
B11	0.05390 (8.9)					
C11	0.11492 (2.1)	y=0.00115x-0.00110 (0.9993)	<1	1:4	<4	
A12	-0.00007 (23)					
B12	0.05472 (0.3)					
C12	0.10800 (1.4)	y=0.00108x+0.00020 (1.0000)	<1	1:4	<4	<4

*the given uncertainty is difference of the showed results, obtained for two sub-samples, analysed in parallel

**Table 4:** Example data of standard addition method for various samples.

Name of solution	Average absorbance for solution (RSD of measurements, %)	Equation of curve in standards addition method (correlation coefficient of the curve)	Result of standard addition method, mg L^−1^	Sample dilution ratio, v:v	Result recalculated for initial sample, mg L^−1^	Final result for sample, mg L^−1^
Reformate						
A11	0.00169 (22)					
B11	0.08216 (0.1)					
C11	0.16784 (2.0)	y=0.00166x+0.00080 (0.9998)	<1	1:4	<4	
A12	0.00107 (7.8)					
B12	0.08199 (1.0)					
C12	0.16549 (2.1)	y=0.00164x+0.00060 (1.0000)	<1	1:4	<4	<4
Izomerizate						
A21	0.00047 (140)					
B21	0.08353 (2.0)					
C21	0.16600 (1.5)	y=0.00166x+0.00060 (1.0000)	<1	1:10	<10	
A22	0.00075 (19)					
B22	0.08199 (0.5)					
C22	0.16580 (1.1)	y=0.00165x+0.00030 (1.0000)	<1	1:10	<10	<10
Ethanol						
A11	0.00001 (45)					
B11	0.01996 (3.2)					
C11	0.04480 (0.9)	y=0.00045x-0.00080 (0.9980)	<1	1:4	<8	
A12	0.00006 (110)					
B12	0.01968 (3.1)					
C12	0.03533 (1.2)	y=0.00045x-0.00070 (0.9984)	<1	1:4	<8	<10
Gasoline, type 1						
A21	0.00613 (13)					
B21	0.06498 (1.7)					
C21	0.13038 (1.2)	y=0.00124x+0.0050 (0.9996)	4.0	1:4	16	
A22	0.00406 (6.3)					
B22	0.06174 (3.1)					
C22	0.12086 (1.1)	y=0.00117x+0.0038 (1.0000)	3.3	1:4	13	15 ± 3*
Gasoline, type 2						
A21	0.01033 (8.9)					
B21	0.08115 (3.1)					
C21	0.14127 (2.4)	y=0.00131x+0.0121 (0.9989)	9.3	1:4	37	
A22	0.01009 (5.4)					
B22	0.07935 (0.7)					
C22	0.14334 (0.8)	y=0.00123x+0.0093 (0.9998)	7.8	1:4	31	34 ± 6*

*the given uncertainty is difference of the showed results, obtained for two sub-samples, analysed in parallel

A difference between two results obtained for a given sample in repeatability conditions (two analysis executed in parallel, using A1-C1 or A2-C2 solutions) should be less than 20% of their average. The final result should be obtained as average value, decreased by blank and multiplied by dilution factor. The content of organic fluorine in an original sample should be reported to the nearest 1 mg L^−1^.

It was found that most of investigated samples do not contain measurable amount of organic fluorine (organic F content below detection limit, 4 or 10 mg L^−1^). However, severe contamination was identified in some cases. For comparison and validation purpose two such samples were analysed using the proposed method and a routine combustion ion chromatography (CIC) [9]. The results of HR-CS FMAS for the two samples were 105 ± 14 and 83 ± 15 mg L^−1^, while the results of CIC were 86 ± 11 and 75 ± 10 mg L^−1^, respectively [1]. Thus, the results of both methods were consistent within their uncertainty, which confirms good accuracy of the proposed method.

## Declaration of Competing Interest

The authors declare that they have no known competing financial interests or personal relationships that could have appeared to influence the work reported in this paper.
